# Availability and Promotion of Cannabidiol (CBD) Products in Online Vape Shops

**DOI:** 10.3390/ijerph18136719

**Published:** 2021-06-22

**Authors:** Eric C. Leas, Natalie Moy, Sara B. McMenamin, Yuyan Shi, Tarik Benmarhnia, Matthew D. Stone, Dennis R. Trinidad, Martha White

**Affiliations:** 1 Herbert Wertheim School of Public Health and Human Longevity Science, University of California San Diego, La Jolla, CA 92093, USA; nhmoy@ucsd.edu (N.M.); smcmenamin@ucsd.edu (S.B.M.); yus001@ucsd.edu (Y.S.); tbenmarhnia@ucsd.edu (T.B.); m3stone@ucsd.edu (M.D.S.); dtrinidad@ucsd.edu (D.R.T.); mmwhite@ucsd.edu (M.W.); 2 Scripps Institution of Oceanography, University of California San Diego, La Jolla, CA 92093, USA

**Keywords:** cannabis, cannabidiol, CBD, e-cigarette, vape, marijuana, hemp, Google

## Abstract

Vaping products containing cannabidiol (CBD), a cannabis-derived compound used in wellness products and available in all 50 US states, were recently implicated in outbreaks of poisonings. Little is known about the commercial availability of CBD products in vape shops (i.e., stores that sell e-cigarettes). To document the availability and marketing of CBD products in online vape shops, in June 2020, we used the Google Chrome browser without cached data to collect the first two pages of search results generated by five Google queries (*n* = 100 search results) indicative of shopping for vaping products (e.g., “order vapes”). We then determined whether and what type of CBD products could be mail-ordered from the returned websites, and whether any explicit health claims were made about CBD. Over a third of the search results (*n* = 37; 37.0%) directed to vape shops that allowed visitors to also mail-order CBD. These shops sold 12 distinct categories of CBD products–some with direct analogs of tobacco or cannabis products including CBD cigarettes, edibles, flowers, pre-rolled joints, and vapes. Two vape shops made explicit health claims of the therapeutic benefits of CBD use, including in the treatment of anxiety, inflammation, pain, and stress. The abundance and placement of CBD in online vape shops suggests a growing demand and appeal for CBD products among e-cigarette users. Additional surveillance on the epidemiology of CBD use and its co-use with tobacco is warranted.

## 1. Introduction

Cannabidiol (CBD) is one of more than 113 identified cannabinoids derived from the cannabis plant. CBD is abundant in the hemp plant, a variety of the *Cannabis Sativa* species that is bred for industrial uses and does not contain the main psychoactive compound in cannabis, ∆^9^-tetrahydrocannabinol (Delta-9-THC). While Delta-9-THC-containing cannabis products remain illegal at the federal level in the United States, the passage of the 2018 Farm Bill provided a legal justification for the manufacturing and sale of “CBD Products” (those containing hemp-derived CBD and ≤0.3% Delta-9-THC) in all 50 US states [1]. Since this time, CBD products have become incredibly popular and so diverse that they can be incorporated into almost every aspect of a consumer’s lifestyle, including bath soaps, candy, cosmetics, drink mixers or tincture, food, medicinal products (e.g., pain pills or sleep aids), pet products, sex lubricants, cigarettes, and vaporizers [2,3].

CBD products have achieved their popularity at least in part because CBD has been widely touted as a cure-all. For example, one cannabis brand *MedMen*^®^ has claimed that CBD can treat acne, anxiety, opioid addiction, pain, and menstrual problems [4]. More recently, CBD has even been promoted as a treatment for COVID-19 in popular news articles [5]. However, the only CBD therapy approved by the US Food and Drug Administration (FDA) is *Epidiolex*^®^, a highly purified form of CBD prescribed to treat Lennox-Gastaut syndrome, Dravet syndrome and tuberous sclerosis complex (3 rare forms of childhood epilepsy) [6,7]. While the pharmacological profile of CBD suggests that additional therapeutic applications of CBD could eventually be approved by the FDA, the public may already perceive that CBD is an effective therapeutic for many health conditions that it may never be approved to treat [8]. This is of particular concern when patients are substituting CBD for other proven effective treatments–prolonging illnesses that could otherwise be alleviated [9].

While commonly dismissed as a benign consumer product, CBD is not devoid of health risks. Known side effects include liver damage, male reproductive toxicity, nausea, vomiting, drug interactions, sedation, and somnolence [6,10]. Moreover, while CBD does not produce the same euphoric experience that Delta-9-THC produce, it interacts with the central and peripheral nervous systems in ways that classify it as psychoactive [11]. For example, CBD is already approved in prescription form to treat certain childhood seizures and other applications are being pursued for the potential to treat anxiety and addiction [12,13]. Given these psychoactive properties, it is possible that CBD could impact individuals’ abilities to operate motor vehicles, but this has not been well studied [14]. Many other health risks and drug interactions that may be associated with adverse events or diminished efficacy of approved therapies could also exist, but in reality, very little is known about the risks of short- or long-term use.

The marketplace of CBD products has also created regulatory complexities that specifically overlap with tobacco regulation and have had public health impacts. CBD and nicotine can be used interchangeably in some vaping devices. This interchangeability can muddle efforts to pin-point culprit products during outbreaks related to vaping products, such as occurred during the e-cigarette or vaping-associated lung injuries (EVALI) outbreak, when many cases used a combination of CBD, nicotine, and Delta-9-THC in their vapes [15]. While at the federal level in the United States, e-cigarette manufacturers are now required to submit product applications to the FDA before they can market and distribute in the United States, the FDA framework for CBD does not require such applications [4]. Without such quality assurance standards, CBD products can be mislabeled and include Delta-9-THC [16] and adulterated CBD products (primarily CBD vapes) have led to mass poisonings [17]. The interchangeability of vaping products can also create loopholes in regulation. For example, one CBD vape brand *bluum* [18] exploits a loophole in nicotine-vaping policy by producing *JUUL*-compatible mango-, berry-, and mint-flavored CBD pod cartridges, even though these flavors were recently prohibited from pod-based e-cigarettes like *JUUL* in the US because of their popularity among youths [19]. Globally, some vape shops have also directly responded to shocks on the marketplace for vaping products by adapting to carry CBD products. For instance, when vaping was temporarily banned in South Africa as a part of COVID-19 emergency response efforts, many vape shops circumvented this ban by switching their product offerings to include CBD e-liquid and vapes [20].

In spite of these challenges and health risks, very little is known about how vape shops (i.e., retailers that sell e-cigarettes/vapes and related components) have responded to the growing popularity of CBD. One in-store surveillance study conducted by Kong et al. found that 6% of vape shops and 47% of combined vape and head shops (i.e., shops that sell drug paraphernalia) in New Hampshire sold CBD- and/or Delta-9-THC-containing products, despite neither CBD- nor Delta-9-THC-containing products being legal in the state at the time [21]. Another in-store surveillance of vape shops across six metropolitan statistical areas (MSAs; Atlanta, Boston, Minneapolis, Oklahoma City, San Diego, and Seattle) conducted by Berg et al. in 2018 identified that 43% of vape shops sold CBD e-liquids and 23% sold other CBD-containing products [22]. An additional phone-based survey of vape shop owners/managers across the same MSAs further substantiated this finding, with 44% of owners/managers reporting that their shops sold CBD e-liquids [23]. Notably, all current surveillance has been limited by its focus on brick-and-mortar retailers, which may not mirror the e-commerce marketplace. Of specific concern with e-commerce is preventing youth purchases, as online retailers have a more limited ability to verify the age of consumers who mail-order their products and youth are often able to successfully bypass age verification to purchase tobacco products including e-cigarettes [24,25].

In this manuscript, we provide e-commerce surveillance on the availability and promotion of CBD products among vape shops returned in typical internet searches indicative of shopping for vaping products. We utilized an existing method that has been previously used to study the availability of mail-order marijuana delivery using the Google search engine [26]. Specifically, we executed a series of Google searches and then reviewed the content of those searches to identify vape shops and further study their product offerings and marketing. Our searching strategy was designed to capture general searches indicative of shopping for vaping products. In a previous analysis of the queries we use, we estimated that there were 30.3 million vape shopping Google searches in 2019 and that the trajectory and growth in these queries followed the trajectory and growth in unit sales of e-cigarettes and had periodicities and holiday patterning that follow the purchasing of other consumer goods [27]. We focused our analysis on the first two pages of search results, because over 80% of Google searchers click a link on the first two pages, with as many as 42% selecting the first link [28]. Among websites returned in this search, we conducted a content analysis to determine whether CBD products could be mail-ordered from the returned website. Since CBD products can come in numerous forms and not just vapes (e.g., capsules, edibles, topicals, and tinctures), we documented the forms of CBD that vape shops allowed visitors to mail-order on their websites. Finally, because CBD products have been marketed extensively with unsubstantiated health claims [4,9], we also conducted a content analysis to determine whether the vape shops made any health claims about CBD products.

## 2. Materials and Methods

We used the Google search engine to obtain a sample of vape shops [26]. Our searching strategy was designed to capture vape shops returned in general searches indicative of shopping for vaping products [27]. We executed Google searches with five shopping indicative terms (i.e., “buy”, “order”, “shop”, “retailer”, or “sale”) in combination with the term “vape” (e.g., “order vape”). Google automatically keyword-stems search terms such that all suffix variations of a keyword are queried as well as alternative verb conjugation. For example, our use of the word “buy” also queries the words “buys”, “buying” and “bought”. All searches were executed in June 2020 in a Google Chrome browser without cached data (e.g., we did not allow location identification). The resulting 100 links (five search term combinations, two pages per search term, and 10 links per page) were retained for further content analysis.

Next, we visited the websites returned in the search results to label whether they allowed visitors to mail-order CBD and also to confirm whether the websites allowed their visitors to mail-order e-cigarettes/nicotine vaping products and components, thereby classifying them as a “vape shop”. An independent review of an overlapping sample of 40 search results suggested very high concordance, with two authors (EL, NM) assigning the same labels on 97.5% of search results for e-cigarettes (Cohen’s k = 0.95) and 97.5% of results for CBD (Cohen’s k = 0.79). Any discordant labels were discussed, and a consensus was reached by unanimous agreement between two authors, EL and NM. For websites that sold CBD products, NM and EL extracted all information about the types of CBD products sold using the categories defined by the vape shop (e.g., “e-liquid”) and then by collapsing these categories into a unifying theme (e.g., “lotions” and “salves” fall under the category “topicals”). We also recorded any explicit claims that CBD treated a health condition (e.g., “treats anxiety”) that were made by the vape shop. We did not investigate claims made by product manufacturers on these websites or other websites discussing the products not included in the vape shops’ page. We focused exclusively on claims for treating medical conditions and do not report vague wellness applications (e.g., “exercise performance”).

We report the number and percentage of unique vape shops that were displayed across the queries and the percentage that allowed visitors mail-order CBD. We also report the page ranking placement of vape shop, which indicates where the website falls in the ordering of search results, with higher results being more likely to be clicked. Among vape shops who sold CBD products, we report the availability of each identified product as a percentage of vape shops and report any explicit health claims made by vape shops. All statistical analyses were performed using R version 4.0.4 (R Foundation for Statistical Computing, Vienna, Austria).

## 3. Results

**Figure 1:**
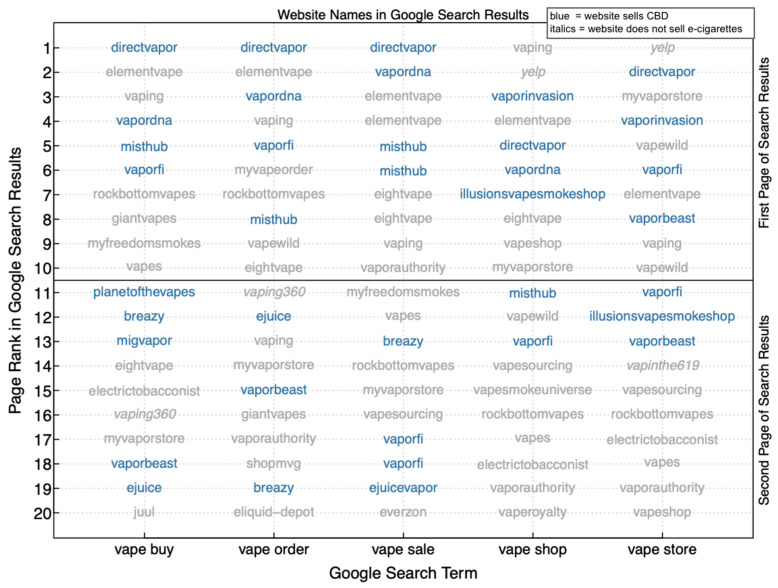
Vape shops who sold CBD products featured prominently in the first two pages of Google search results indicative of shopping for vaping products, June 2020; Notes: Blue text indicates visitors could purchase CBD products from the webpage. Three websites (indicated in italics: Yelp, vaping360, & vapinthe619) did not allow visitors to mail-order e-cigarettes. CBD = Cannabidiol.

Thirty-two unique vape shops were represented across searches indicative of shopping for vaping products (Figure 1). Other search results included duplicate links to the same vape shop as well as websites that did not sell vaping or CBD products, including websites for a brick-and-mortar vape shop, a crowdsourcing website for brick-and-mortar retailers, and a website that offers reviews of e-cigarettes/vaping products. Among all 32 vape shops that provided mail-order purchasing of CBD products or e-cigarettes, 12 (37.5%) provided both mail-order CBD and e-cigarettes, 20 (62.5%) provided only mail-order e-cigarettes. Figure 1 also illustrates the distribution of vape shops across the respective search results. Vape shops that sold CBD were represented in 37 of the 100 search results (37.0%) and ranked highly in the search results, with 3 of the 5 first-linked and 20 of 50 first-page results linking to vape shops that sold CBD.

**Figure 2:**
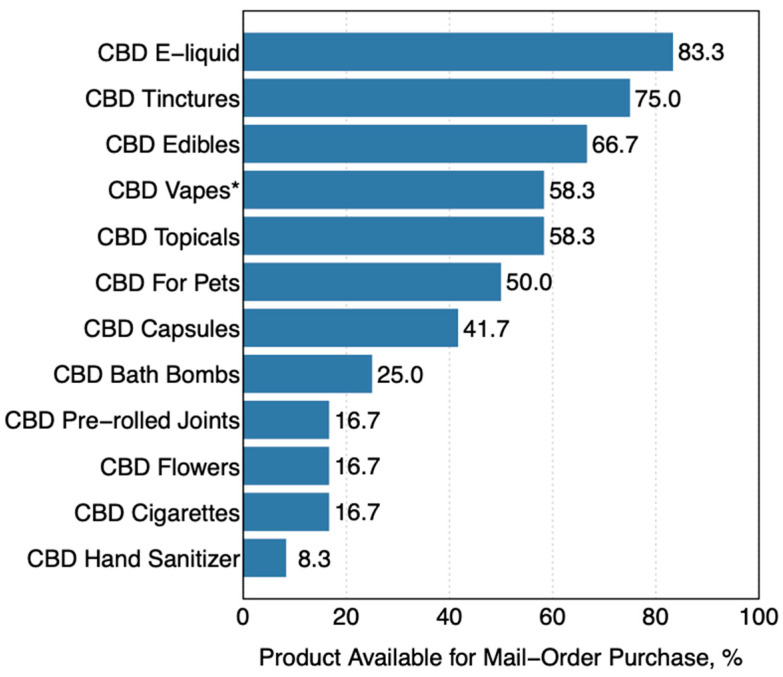
The type of CBD products offered at vape shops appearing the first two pages of Google search results of queries indicative of shopping for vaping products, June 2020, *n* = 12; Note: * CBD vapes refers to self-contained CBD vaping kits with pre-filled cartridges and vapes as well as disposable devices. CBD = Cannabidiol.

We identified 12 distinct categories of CBD products offered in the 12 vape shops that sold mail-order CBD. Among these 12 vape shops, e-liquid was the most commonly offered category of CBD products (Figure 2), offered by 10 of the vape shops (83.3%), followed by tinctures (*n* = 9; 75.0%) and edibles (*n* = 8; 66.7%). The next most frequent were CBD topicals (*n* = 7; 56.2%); self-contained CBD vaping products, which includes CBD vaping kits with pre-filled cartridges and disposable devices *(n* = 7; 56.2%); CBD pet products *(n* = 6; 50.0%); CBD capsules/pills *(n* = 5; 41.7%); CBD bath bombs (*n* = 3; 25.0%); CBD cigarettes (*n* = 2; 16.7%); CBD pre-rolled joints (*n* = 2; 16.7%); CBD flowers (*n* = 2; 16.7%); and CBD hand sanitizer (*n* = 1; 8.3%). Two of the 12 vape shops did not offer CBD vaping products, but instead offered CBD capsules, CBD edibles, CBD for pets, and CBD tinctures; and CBD edibles, CBD tinctures, and CBD topicals, respectively.

Two of the 12 vape shops that sold CBD made explicit health claims of unsubstantiated therapeutic benefits of CBD. These are presented in Table 1.

## 4. Discussion

This content analysis indicated that over a third of the search results in the first two pages of Google search results indicative of shopping for vaping products directed to vape shops that sold and promoted CBD products. While CBD vaping products were unsurprisingly the most commonly available product, these vape shops also offered other types of CBD including CBD cigarettes, edibles, topicals, and pet products. Several retailers also made explicit health claims about CBD that have not been substantiated and are not FDA-approved for recommended uses of CBD.

The prevalence of CBD co-marketing in our analysis of online vape shops mirrors the findings reported from in-store surveillance of brick-and-mortar vape shops and phone surveys with owners and managers of these shops, with all data suggesting that ~40% of both online and traditional vape shops have expanded their product offerings to include CBD [21,22,23]. Our analyses suggests that product offerings were not limited to vaping-type CBD products, with 12 distinct product categories identified. Some products were direct analogs of tobacco or cannabis products, including CBD cigarettes, edibles, flowers, pre-rolled joints, and vapes. This finding adds to one other study that identified growing sales of CBD vapes, cigar wraps and smokeless papers in Nielsen retail scanner data [29]. The prevalence of these products in vape shops suggest that many e-cigarette and/or tobacco users may be interested in trying CBD products, but this cannot be confirmed because of a dearth of information on the epidemiology of CBD use, let alone its co-use with tobacco [11]. For example, there are no population surveys of CBD use. Measures of CBD use and reasons for use should be integrated into current substance use surveillance efforts.

We identified two vape shops that made explicit health claims about CBD’s effectiveness for treating anxiety, inflammation, pain, and stress, which are not FDA-approved uses of CBD. The claims we identified are consistent with findings of a recent analysis of user testimonials on the largest social media forum devoted to discussing CBD that found that psychiatric (e.g., anxiety) and orthopedic (e.g., joint pain) conditions were the most commonly reported medical reasons for using CBD [9]. Such unsubstantiated claims may increase use of CBD products, prolong illnesses with existing effective treatments, and expose consumers to both known and unknown health risks without conferring any of the purported benefits. For this reason, the FDA prohibits the sale of CBD products with such marketing claims, and has already sent warning letters to some companies that have marketed CBD in this manner [30]. While we could not assess this in our methodology, it is possible that retailers’ knowledge of the FDA’s stance and actions on marketing CBD with health claims may at least partially explain why only two vape shops that sold CBD made health claims rather than all of them. Moreover, aside from warning letters, in congressional testimony, the FDA commissioner stated that the agency would take stronger and wider-ranging actions if patients with diagnosable conditions were using CBD as a substitute or adjunct for approved therapies [31]. While preliminary evidence suggests many consumers are indeed using CBD as a substitute or adjunct, additional population-based surveillance is needed to monitor how and why consumers are using CBD and promotional efforts that lead consumers to use CBD in this manner.

Continued monitoring of this online marketplace for vaping products will also be needed to understand how e-commerce will change with the passage of the Preventing Online Sales of E-Cigarettes to Children Act in the US scheduled to be implemented 120 days after the bill’s enactment on 27 December 2020 [32]. This Act will prevent the mailing of e-cigarettes via the US Postal Service and requires an in-person ID check upon delivery of online orders that arrive via other couriers. These changes could disrupt the economics of mail-order vaping. Although the bill defines included products as “any electronic device that, through an aerosolized solution, delivers nicotine, flavor, or any other substance to the user inhaling from the device”; it also exempts any products that the FDA has approved for any “therapeutic purpose”. Given that some websites are already making therapeutic claims about CBD products even though this is not allowed by the FDA, it seems possible that vape shops may incorrectly assume that this language exempts CBD vaping products from the policy. As a contemporary example of this, many vape shops in South Africa circumvented the temporary ban on vaping products in South Africa that was a part of emergency efforts for COVID-19 by switching their product offerings to include CBD products including vapes [20].

Our analysis of shopping queries was limited in that we could not determine how often queries would result in purchases, and the quantity and type of products purchased. Further, some searches may be unrelated to seeking e-cigarette retailers, and some retailers may be illegitimate or scams. However, aside from their face validity, nearly all searches directed to retailers who sold e-cigarettes. A previous analysis of trends in these searches also suggested they followed the trajectory and growth in unit sales of e-cigarettes and had periodicities and holiday patterning that follow the purchasing of other consumer goods (e.g., elevated in summer months and spikes around the US Thanksgiving holiday) suggesting that they are correlated with purchasing behaviors including e-cigarettes [27]. Additionally, while we used a browser without cached data to allow other researchers to replicate our search strategy, an exact replication cannot be performed as searching Google from a historical date (in our case June 2020) is not possible. It is also unclear how common this strategy of searching for vaping products without cached data is (compared to searching with cached data) and it is impossible to determine exactly whether searches that are tailored to each individual’s browser data and location would return similar results. Fortunately, CBD availability in vape shops in other locations or countries can easily be determined by replicating our search strategy (e.g., “order vapes”) and modifying the settings for other locations of interest.

## 5. Conclusions

Limitations notwithstanding, our results have important implications for public health research and practice. In the context of tobacco control, the positioning of CBD products in vape shops could erode the impact of tobacco control efforts. E-cigarette users could begin using these products under the false premise that they are risk free when they are not or using them therapeutically for applications that are not proven. CBD product manufacturers are also exploiting loopholes in vaping policy (e.g., making tobacco-like products or using explicit health claims for CBD) that in their absence might otherwise result in cessation from the vaping of any substance or prevent initiation. The abundance and placement of CBD in vaping search results also underscores a need for more information on the epidemiology of CBD use and co-use with tobacco.

## Figures and Tables

**Table 1:** Explicit health claims made by the vape shops that appeared in the first two pages of Google search results of queries indicative of shopping for vaping products, June 2020.

Index	Explicit Health Claims
1	“incredibly helpful and healing…for many different situations. Its physical effects include: anti-inflammatory qualities, pain, and stress relief”
2	“the best hemp oil for pain, anxiety, inflammation, or whatever purpose is going to be the best hemp oil period” (sic)

## Data Availability

Data are freely available by using the search terms we provided in the methods on any Google browser session or by visiting any of the websites we provided in the analyses.
